# Flexible Screen Printed Aptasensor for Rapid Detection of Furaneol: A Comparison of CNTs and AgNPs Effect on Aptasensor Performance

**DOI:** 10.3390/nano10061167

**Published:** 2020-06-15

**Authors:** Ali Douaki, Biresaw Demelash Abera, Giuseppe Cantarella, Bajramshahe Shkodra, Asma Mushtaq, Pietro Ibba, AKM Sarwar Inam, Luisa Petti, Paolo Lugli

**Affiliations:** Faculty of Science and Technology, Free University of Bolzano-Bozen, 39100 Bolzano, Italy; BiresawDemelash.Abera@natec.unibz.it (B.D.A.); Giuseppe.Cantarella@unibz.it (G.C.); Bajramshahe.Shkodra@natec.unibz.it (B.S.); Asma.Mushtaq@natec.unibz.it (A.M.); Pietro.Ibba@natec.unibz.it (P.I.); AKMSarwar.Inam@natec.unibz.it (A.S.I.); Paolo.Lugli@unibz.it (P.L.)

**Keywords:** aptamer, furaneol, aptasensor, carbon nanotubes, silver nanoparticles, biosensor

## Abstract

Furaneol is a widely used flavoring agent, which can be naturally found in different products, such as strawberries or thermally processed foods. This is why it is extremely important to detect furaneol in the food industry using ultra-sensitive, stable, and selective sensors. In this context, electrochemical biosensors are particularly attractive as they provide a cheap and reliable alternative measurement device. Carbon nanotubes (CNTs) and silver nanoparticles (AgNPs) have been extensively investigated as suitable materials to effectively increase the sensitivity of the biosensors. However, a comparison of the performance of biosensors employing CNTs and AgNPs is still missing. Herein, the effect of CNTs and AgNPs on the biosensor performance has been thoughtfully analyzed. Therefore, disposable flexible and screen printed electrochemical aptasensor modified with CNTs (CNT-ME), or AgNPs (AgNP-ME) have been developed. Under optimized conditions, CNT-MEs showed better performance compared to AgNP-ME, yielding a linear range of detection over a dynamic concentration range of 1 fM–35 μM and 2 pM–200 nM, respectively, as well as high selectivity towards furaneol. Finally, our aptasensor was tested in a real sample (strawberry) and validated with high-performance liquid chromatography (HPLC), showing that it could find an application in the food industry.

## 1. Introduction

Furaneol (4-Hydroxy-2,5-dimethyl-3(2H)-furanone) is an important flavoring agent, which can be naturally found in different products, such as strawberries or thermally processed foods [1]. Moreover, the compound has specific physiological properties, such as anti-infective activity (is capable of inhibiting the spread of infectious organisms or killing infectious organisms) during microbial infections in humans, protection of human erythrocyte membranes and lipoproteins against iron-induced oxidative modifications, as well as inhibition of hyperpigmentation [2]. On the other hand, recently, extensive studies on the cytotoxicity of furanone compounds have been done, where the results showed that furanone compounds have a mutagenic, cytotoxic, and DNA-breaking activity effects [3,4]. Furthermore, furaneol detection has many potential applications in the food industry, such as process control during food production [5]. Also, it could be used for the certification of food products origin and control of beverage quality [6,7]. Moreover, controlling the ripeness of the strawberry could also be another interesting application [8]. Furaneol is mostly detected using well-established but time-consuming techniques, such as gas chromatography [7,8,9,10,11]. Therefore, developing a sensor for cheap, fast, and selective detection of furaneol is of uttermost importance for food industry. In this context, biosensors are extremely appealing, due to the possibility of reducing fabrication cost and detection time. A biosensor is an analytical device that combines a biological component (the so-called ‘biorecognition element’) with a physicochemical detector called a ‘transducer’ to detect specific chemical substances [12]. Among different biorecognition elements, aptamers are characterized by different advantages such as low cost, facile synthesis, thermostability, and shelf life [13]. Hence, the aptasensors have become attractive in the bioanalytical field for different reasons: simplicity, high sensitivity, high selectivity, low cost, and fast response [14,15,16], compared to more traditional biorecognition elements like enzymes and antibodies [17]. Aptamers are in vitro-selected single-stranded DNA or RNA that are isolated via an in vitro selection process called ‘systematic evolution of ligands by exponential enrichment’ (SELEX) [18]. Aptamers are capable of binding specifically with target molecules such as cell surfaces, small molecules, and proteins with high selectivity, due to their three-dimensional folding [19]. In the absence of the analyte, the redox label is distant from the electrode surface, thus generating low output current. In the opposite case, in the presence of the analyte, the latter binds with the aptamers and undergoes a conformational change bending closer to the electrode surface [20], thereby increasing the current. In this way, the current variation can be directly and selectively correlated to the concentration of the analyte.

To increase the sensitivity of the biosensor, usually, the working electrode is functionalized with nanomaterials such as carbon nanotubes (CNTs) and metal-nanoparticles (e.g., gold, platinum, silver, titanium, and iron) [14]. CNTs have attained great interest in electrochemical biosensors due to their high surface area to volume ratio and stability [21], as well as due to their good conductivity. Usually, CNTs are used to increase the electron transfer between the electrolyte and the surface of the electrode [15,16,22,23]. Besides CNTs, metal nanoparticles (MNPs) [24,25] are also broadly used in electrochemical sensors due to their small dimensional scale, good stability, and excellent conductivity [25]. Although CNTs and MNPs have been extensively studied in biosensors, to the best of our knowledge, a comparison among the performance of biosensors employing them is still missing. Moreover, currently, there is considerable interest in using aptamers (DNA, RNA, or peptides) in gas sensing [26]. Hence deposition of the nanomaterials to increase the sensitivity will be employed. However, using CNTs could affect the measurement (because of the interaction between CNTs and gases) [27]; therefore, finding an inert alternative to CNTs could find an application in the future. The aim of this work is the fabrication of an electrochemical aptasensor for fast and selective detection of furaneol, as well as the study of the impact of different electrode functionalization’s with CNTs and AgNPs on the device performance. To achieve this, electrodes were screen-printed using silver (Ag) and silver chloride inks (AgCl) and then functionalized with two different nanomaterials (CNTs or AgNPs) (to increase the current transfer and the electroactive surface area). In this work, the experimental conditions were first optimized to improve analytical performance; next, a series of concentrations (1 fM–40 µM) of the analyte of interest (Furaneol) were used to test the performance of the aptasensors. Based on the results obtained, the CNT-modified electrode (CNT-ME) aptasensor showed a lower limit of quantification (LOQ) of 1 fM as compared to 2 pM of the AgNPs-modified electrodes (AgNP-ME) aptasensor. This improvement was attributed to the high electroactive area of CNTs (1.79 cm^2^) as compared to 0.47 cm^2^ and 1.01 cm^2^ of bare electrode and AgNP-ME, respectively. Due to the better performance, CNT-ME aptasensors were carefully evaluated in terms of linear range (1 fM–35 µM), selectivity, regeneration, high reproducibility with relative standard deviation (RSD) of 3.16%, stability over time (25 days at 4 °C), and mechanical bendability. Furthermore, our aptasensors showed a rapid response, requiring only 10-min incubation of the sample solution before measurements. Finally, our devices were validated with an HLPC by measuring furaneol concentration in real samples (Elsanta strawberry), showing good accuracy of 5–12%.

## 2. Materials and Methods

### 2.1. Materials and Reagents

4-Hydroxy-2,5-dimethyl-3(2H)-furanone, 4,5-dimethyl-3-hydroxy-2,5-dihydrofuran-furanone (sotolon), 5-ethyl-4-hydroxy-2-methyl-3(2h)furanone (homofuraneol), 5-ethyl-3-hydroxy-4-methyl-2(5h)-furanone (maple furanone), magnesium chloride anhydrous, hydrochloride acid, Tris, sodium chloride, cysteamine, N(3-dimethylaminopropyl)N ethylcarbodiimide (EDC), N-Hydroxysulfosuccinimide sodium salt (NHS), 1,8-Octanedithiol, N-Hydroxy succinimide (NHS), ethanol, 11-mercaptoundecanoic acid (11-MUA), phosphate buffer saline (PBS), potassium chloride (KCl), potassium ferricyanide III (K_3_[(Fe(CN)_6_]), potassium ferricyanide II trihydrate (K_4_[(Fe(CN)^6^]x3H_2_O), Celite 545, MWCNT carboxyl, sodium dodecyl sulphate (SDS) were obtained from Sigma Aldrich (Munich, Germany). Furaneol aptamer (5′-amine group -CGCCAGCTCATTCCTCACCACGAGAAAGGAGCTCGATGAACTGCGAGCCGGATTCGACCCTATGCGAGTAGGTGGT- methylene blue-3′) amine group at the 5′-end was ordered from Microsynth AG (Balgach, Switzerland) [19]. Polyethylene Terephthalate (PET) flexible substrate with a 125-micron thickness was purchased from Mylar (Chester, VA, USA). Ink pastes, silver chloride ECI 6038E and silver ECI 1011, were purchased from LOCTITE E&C (CA, USA). All chemicals used in this work are analytical grade and were used without any further purifications.

### 2.2. Fabrication of the Aptasensor

#### 2.2.1. Printing the Electrodes

The sensor was fabricated by screen-printing (automatic screen-printing machine -Aurel C920-, Italy) of the electrodes on a flexible PET substrate using Ag/AgCl based polymeric ink pastes. Figure S1 shows a screen-printed flexible electrode, consisting of an Ag working electrode (WE), an AgCl reference electrode (RE), and an Ag counter electrode (CE), with a total length of 22 mm and a width of 8 mm. The sensor was produced as follows. First, WE, CE, and the lower half part of the RE were screen-printed and cured at 120 °C for 15 min. Then, the upper half of the RE was screen-printed and cured at 120 °C for 15 min. Finally, a passivating layer was screen-printed on top of the electrodes to isolate the conductive area, to contain the analyte droplet, and to have a reproducible working area. After the fabrication, the screen-printed electrodes were ultrasonically cleaned in ethanol and ultrapure water for 5 min each, respectively. Afterwards, a two-step electrochemical cleaning was performed first under a basic condition in 3M NaOH and a potential ranging from −0.35 V to −1.35 V; then under an acid condition (0.5 M H_2_SO_4_) and a finally potential ranging from −0.35 V to −1.5 V., Both scans were performed until a stable cyclic voltammogram was obtained. 

#### 2.2.2. Preparation of Nanomaterials 

A dispersant solution was prepared by dissolving SDS 0.5 wt% in deionized (DI) water, followed by 1 h stirring. Then, 0.05 wt % of CNT-COOH were added in the dispersant SDS solution. Next, horn sonication for 30 min (30% power) (Fisher brand Q500) and centrifugation for 120 min at rpm and rotor radius was 2.5 cm [28] was conducted, keeping the supernatant. Finally, the dispersion was mixed with a solution mixture of 300 mM EDC and 35 mM NHS (pH 6.0) (1:1 v/v) to activate the COOH group. Silver nanoparticles solution was obtained by dilution of 0.05 wt% in solution with triethylene glycol monomethyl ether.

#### 2.2.3. Stepwise Fabrication Strategy

##### CNT-Modified Electrode (CNT-ME)

**Figure 1:**
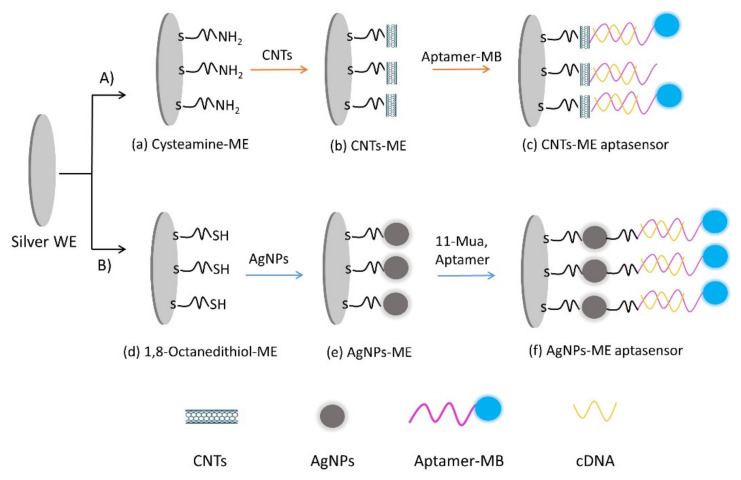
Fabrication process flow of CNT-ME (**A**) and AgNP-ME (**B**) aptasensors: (A) first bare electrode was modified with cysteamine (**a**), and CNTs were immobilized on top of the cysteamine-ME to form CNT-ME (**b**); afterward, aptamer-MB was immobilized onto the CNT-ME (**c**). (B) first bare electrode was modified with 1,8-octanedithiol (**d**); afterward, AgNPs were immobilized on top of bare-1,8-octanedithiol to form AgNP-ME (**e**). The AgNP-ME was modified with 11-MUA, and finally, aptamer-MB was immobilized onto the 11-MUA-AgNP-ME (**f**).

The freshly sonicated and electrochemically cleaned Ag electrodes were immersed in 1 mM cysteamine (has a thiol group on one side and an amine group on the other side) for 24 h. Cysteamine will chemically attach to the silver electrode due to the strong interaction between the noble metals and the thiol group. Leaving the amine group free (Figure 1a). Next, the CNT solution was drop-casted onto the WE for 5 h (Figure 1b). The carboxylic group of the CNTs formed a chemical bond with an amine group of cysteamine-ME, then immersed in DI water for 10 min to remove the SDS. Then, 10 µL of a mixture of 1 µM aptamers, 300 mM EDC and 35 mM NHS (pH 7.0) was drop-casted and air-dried for 2 h (Figure 1c). Afterward, the aptamer was hybridized with 20 µM cDNA over 2 h (unlike the overtime stability test, the aptasensors were stored without cDNA and were hybridized with cDNA before the measurement), followed by washing off the excess aptamer with DI water, and then keeping the aptasensors at 4 °C until they were ready for use. Figure 1A is showing the CNT-ME complete immobilization procedure.

##### AgNPs Modified Electrode (AgNP-ME)

Mixed self-assembled monolayers (SAMs) were prepared by immersing the freshly cleaned silver screen-printed electrodes (SPEs) in an ethanol solution containing 10 mM 1,8-octanedithiol (has two thiol groups on both ends) for 24 h, One of the thiol groups will form a chemical bond with the silver electrode, which led to the formation of a monomolecular layer SH-SAM on top of the working electrode with a free thiol group which will be used to anchor the AgNPs onto (Figure 1d). Afterward, 1,8-octanedithiol residual molecules were removed by washing the electrode surface with a large amount of ethanol and water and subsequentially drying it using an air gun. The prepared SH-SAM electrodes were then covered with a colloidal solution of AgNPs solution (average size: 50 nm) for 30 min. Due to the strong interaction between the noble nanoparticles and the thiol molecule, the AgNPs reacted with the S-H end of 1,8-octanedithiol and hence led to the immobilization of the AgNPs onto the electrode (Ag-SAM-AgNPs) (Figure 1e). The unbound nanoparticles were removed by washing with deionized water. After that, the Ag-SAM-NPs electrodes were immersed in 1 mM 11-MUA for 24 h to form the SAM-COOH layer. Afterwards, the Ag-SAM-NPs electrodes were immersed in 1 mM 11-MUA (has a thiol group on one side and a carboxylic group on the other side) overnight, where the terminal -SH group of 11-MUA was attached to AgNPs, leaving a free carboxylic group to be attached covalently with the amine group at -5′ end of the aptamers. Furthermore, 10 µL of 1 µM aptamers, 300 mM EDC, and 35 mM NHS pH 7.0 drop-casted on top of the working electrode and air-dried for 2 h (Figure 1f). Finally, the aptamer layer was hybridized with 20 µM cDNA 5′-CATCGAGACTCC-3′, over 2 h and then kept at 4 °C until use. The schematic of the fabrication of the AgNPs modified electrode (Ag-SAM-AgNPs-aptamers-MB) is shown in Figure 1B.

### 2.3. Electrochemical Characterization of Furaneol Aptasensor

Cyclic and square wave voltammetry measurements were performed using a source meter (KEITHLEY 2614B Source Meter ^®^, a Tektronix Company, USA). By covering the three electrodes (WE, CE, and RE) with 50 µL of 1 mM [Fe (CN)_6_]^3–/4–^ containing 0.1 M KCl solution, at a scan rate of 100 mV/s and a scan potential between −1 to 1 V. Electrochemical impedance spectroscopy (EIS) measurements were carried out with a Keysight impedance analyzer E4990a, with an AC amplitude of 50 mV in a frequency range from 20 Hz to 100 kHz and a sampling rate of 100 points.

### 2.4. Optical Characterization

A 3D-optical profilometer (ProFilm3D from Filmetrics, Unterhaching, Germany), was used to measure the roughness of the electrodes and the aptasensors.

### 2.5. Detection of Furaneol

The final fabricated aptasensor was incubated in the furaneol solution containing a selection buffer (SB): (2 mM MgCl_2_, 100 mM NaCl, 5 mM KCl, 1 mM CaCl_2_, 20 mM Tris-HCL (pH 7.6)) at room temperature. Then, the aptasensor was washed with PBS buffer to remove the unattached analyte and cDNA. Afterward, square wave voltammetry (SWV) analyses were performed by covering the electrodes with 50 µL 0.1 M of PBS (pH 7.4), using the following parameters: scanning ranges from 0 to −0.5 V, amplitude of 50 mV, step potential of 25 mV and frequency of 50 Hz. The MB oxidation peak was obtained at −0.25 V, where the height of the current peak was recorded. All electrochemical measurements were performed in five replicates. The aptasensor could be reversed to its initial state by removing the analyte bound to the aptamer by immersion in urea solution (8 M) for 10 min, followed by washing with DI water.

### 2.6. Mechanical Characterization

The aptasensor performance under mechanical stability was characterized under bending deformation using a custom-made cyclic bending setup. Using two parallel clamps (one fixed and one movable) and controlled with a LABVIEW program 2017 (NI, TX, USA) (Figure S2), the aptasensor was mounted in the clamps. Afterward, it was flattened and bent down to 6 mm of bending radius for multiple cycles. The performance of the device was evaluated in the detection of 1 µM of furaneol after a certain number of cycles (100, 500, 1000, 1500, 2000, 3000 cycles). Before mounting the sample again in the bending system, it was regenerated by immersing in urea (8 M) for 10 min.

### 2.7. Detection of Furaneol in a Real Sample and Validation with HPLC

Furaneol can naturally be found in many fruits, specifically in strawberries, with a concentration-dependent on the maturation stage. In this way, by measuring the concentration of furaneol in strawberry, it is possible to confirm the ripening stage of the fruit [29]. Therefore, to confirm the reliability of the constructed aptasensor, we grew strawberry plants (Elsanta strawberry) in a climate chamber under controlled temperature and humidity (T = 24 °C and RH = 70%). Fruits (n = 3) at different maturity stages were collected, and their content of furaneol was quantified using the developed aptasensor and high-performance liquid chromatography (HPLC), subsequentially comparing the results.

#### 2.7.1. Characterization of Fruit Maturation Stages

The fruit maturation stages were quantified in terms of color using a hand-held Spectrophotometer (Chroma Meter CR-400, Konica Minolta Corp., Osaka, Japan). For the color, L is the lightness (black (0) and white (100)), a is the color difference between green and red, and finally, b represents the color difference between yellow color and blue color. L, a, and b scores were the mean of five random measurements. 

#### 2.7.2. Sample Preparation

For the preparation of the real samples for HPLC and the aptasensor measurement, strawberries were cut into small pieces. Pieces from three different fruits from each maturation stage (unripe, during ripening, and ripe), were randomly sampled and ground with 5 mL of distilled water at room temperature. Whatman filter paper was first added on top of Ceramic Porcelain Buchner Chemistry Lab Filter (Haldenwanger, Erlangen, Germany), and then Celite 545 (10 g) was added on top of it. Finally, covered with another Whatman filter paper so that pouring the sample did not disturb the surface of the Celite 545. After that, the filtration system was wetted first with distilled water. The grounded, real sample was filtered, then washed three times with 10 mL of distilled water. Subsequentially, the resulting solution was filtered again first through a 0.45 µm and then through a 0.2 µm nylon membrane before HPLC analysis. Finally, the real sample was diluted, and then the measurement results were multiplied by the dilution factor.

#### 2.7.3. HPLC Analysis

Furaneol content was analyzed using 1525 Waters HPLC (Waters Corporation, MA, USA) equipped with a binary pump, an auto-sampler injection system, a Symmetry C18 Column (2.1 × 50 mm, 3.5 µm, Waters Corporation, MA, USA) and a photo-diode array detector (PDA 2998) set at 286 nm. Shimadzu Chem Station for Windows (Shimadzu Technologies) was used to control the system. The resultant from the sample preparation was subjected to HPLC analysis. The mobile phase consisted of methanol/0.5% formic acid solution (v/v), and the following methanol flow gradient: 15/85 for 2 min, 50/50 for 24 min, 100/0 for 27 min, 100/0 for 29 min, and 15/85 for 33 min [30]. The chromatographic separation was conducted out at a flow rate of 0.8 mL/min for 40-min, and injection volume of 20 μL. The furaneol standards (Sigma-Aldrich) was used to prepare the calibration curve (0.1–2.5 mM, R2 = 0.9964). The samples were injected in duplicate.

## 3. Results and Discussion

### 3.1. Working Principle of the Aptasensor

**Figure 2:**
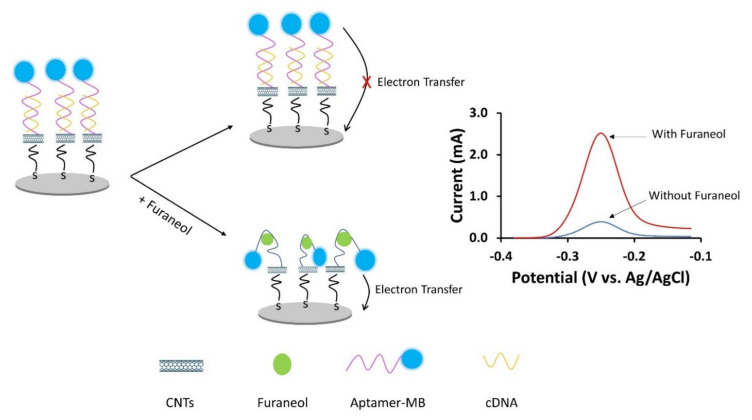
Schematic diagram of the working principle of the CNT-ME aptasensor for furaneol detection by using a carbon nanotubes electrode modified with aptamer-MB and cDNA. The current change generated by the MB label was used for furaneol detection.

The schematic, working principle of the CNT-ME aptasensor, is shown in Figure 2. Furaneol aptamers, labeled with methylene blue MB on the 3′-end and amine group at the 5-end, were immobilized on a silver electrode. In the absence of furaneol, the MB-aptamers molecules formed a rigid structure with cDNA, and thus the redox label (MB) was distant from the electrode surface; hence low current was generated. In the presence of the analyte, the latter binds with the MB-aptamers molecules, because of the structure-switch between the capture probe and the target [19]. Then the aptamers underwent a conformational change and bent closer to the electrode surface [31,32]; thus, the current generated was increased.

### 3.2. Characterization of Stepwise Fabrication of the Aptasensor

#### 3.2.1. Surface Characterization

The surface roughness (R_q_) of the electrodes before and after the immobilization of the nanomaterials and the aptamers were measured by ProFilm3D. The Rq of the bare electrode was 3.12 µm (n = 5), after the immobilization of CNTs and AgNPs and aptamers, the surface roughness of CNT-ME and AgNP-ME was increased to 3.23 µm and 3.35 µm, respectively. This may be due to the immobilization of the nanomaterials [33,34].

#### 3.2.2. Electrochemical Characterization

To monitor the fabrication process of the aptasensor and to check if the CNTs, AgNPs, and the aptamers were properly immobilized on the surface of the electrode, EIS and CV were applied to investigate the effect of the nanomaterials and aptamers immobilization on the electron transfer and the electrode surface resistance. FTIR was performed after each step to monitor the chemical bonding formation. 

**Figure 3:**
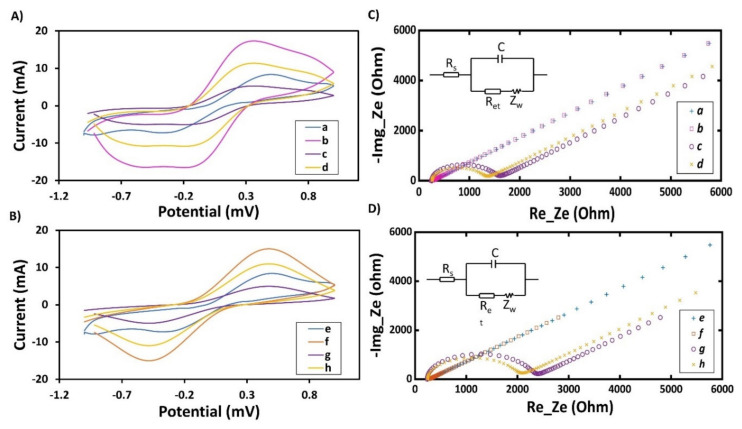
(**A**) and (**B**) CV curves, (**C**) and (**D**) EIS spectra of the stepwise modified electrode in 5 mM [Fe(CN)6]3/4- aqueous solution containing 0.1 M KCl: (**a**) bare (in blue), (**b**) bare/AgNPs (in fuchsia), (**c**) bare/AgNPs/MB-aptamers (in purple), (**d**) bare/AgNPs/MB-aptamers/cDNA (in yellow), (**e**) bare (in blue), (**f**) bare/CNTs (in orange), (**g**) bare/CNTs/MB-aptamers (in purple), (**h**) bare/CNTs/MB-aptamers/cDNA (in yellow).

In CV, the oxidation and reduction current peaks are helpful to characterize the different MEs. Figure 3A,B show a pair of well-defined redox peaks that were recorded for bare electrode and after the immobilization of the nanomaterials and aptamers for AgNP-ME and CNT-ME, respectively. The bare electrode redox peak (5 mA) is related to the high electron transfer between the [Fe (CN)_6_]^3–/4–^ in the electrolyte and the electrode surface, and this value is higher than the ones recorded for gold or carbon electrodes due to the better conductivity of silver ink [35]. This high electron transfer will enhance the sensitivity of the aptasensor. For CNT-ME, after CNTs deposition, the peak current was increased (I_p_ = 15.02 mA) due to the increased electroactive area and to the good conductivity of the CNTs, which promoted the electron transfer between the electrolyte and the electrode [14]. Furthermore, the immobilization of the aptamers onto the CNTs led to a dramatic decrease (I_p_ = 4.92 mA) in the redox current peak, which is in agreement with previous work [36,37]. This may be due to the DNA backbone, which is composed of phosphate groups and sugars. The phosphate groups are negatively charged, which makes the aptamers negatively charged; thus, the immobilized aptamers repel the [Fe (CN)_6_]^3–/4–^ anions from the electrode surface and also acts as a barrier for electron transfer [37]. After incubation of the CNT-ME/aptamer with cDNA, the redox current peak was slightly increased (I_p_ = 10.94 mA), which may be due to the hybridization that makes the negatively charged Aptamer-MB distant from the electrode surface, hence increasing electron transfer near the electrode surface [38]. For the AgNP-ME, immobilizing the AgNPs also increased the peak current, which resulted in being higher compared to the CNT-ME, due to the higher conductivity of AgNPs compared to CNTs. After immobilizing the aptamers onto the AgNP-ME, the peak current was decreased; however, this decrease was lower than the CNT-ME/aptamer. This may be due to the high electroactive area of CNT-ME compared to the AgNP-ME one, thus led to immobilization of a higher amount of aptamers and the repel of [Fe (CN)_6_]^3–/4–^ anions was higher [21].

EIS is a well-known tool used for monitoring the interfacial change of the electrode surface. Figure 3C,D illustrate typical Nyquist plots performed after the deposition of each layer (nanomaterials and aptamers). To relate the biological and the electrical domains and to explain the impedance output, the Randles equivalent circuit (in the inset in Figure 3C) was used, as this is designed to provide the best fit of the impedance output [39]. In this model, C is the capacitance created between the electrode surface/electrolyte. The Warburg element (Z_w_) was added to the equivalent circuit to model the mass diffusion process of the anions in the electrolyte (bulk) towards the electrode surface. R_s_ is the electrolyte resistance, whose values were almost constant (244–250 Ω), due to the use of the same electrolyte during the EIS experiments. R_et_ is related to the electron transfer between the electrolyte and the electrode surface, which is represented with the diameter of the semicircle at high frequencies. The bare electrode exhibits a very small R_et_ value (43 Ω) (Table S1), suggesting a low electron transfer resistance. Furthermore, the addition of CNTs led to an almost straight line with a decrease of the R_et_, indicating that the electron transfer resistance got smaller due the fact that the nanomaterials promote electron-transfer between the electrode surface and the electrolyte; where the R_et_ after assembling the AgNPs was smaller and this due to the high conductivity of AgNPs conductivity compared with that of CNTs. Immobilizing the aptamers on CNT-ME and AgNP-ME led to an increase in the R_et_ to 1956 Ω and 1238 Ω, respectively, because of the electrostatic repulsive interaction between the negatively charged phosphate groups (the backbone of the DNA) of the aptamers and [Fe(CN)_6_]^3–/4–^ anions [40]. Thus, the aptamers acted as a barrier between the electrolyte and the electrode surface. Moreover, the R_et_ registered from CNT-ME was higher compared to the AgNP-ME one, maybe due to the higher electro-reactive area of CNT-ME compared to the AgNP-ME. Indeed, with a higher area to volume ratio, a higher number of aptamers on the CNT-ME are bond, consequently increasing the surface resistance. After the addition of cDNA, the aptamers got distant from the electrode surface, which increased the electron transfer, hence reducing the surface resistance.

#### 3.2.3. FTIR Characterization

Figure S3 shows the infrared (IR) spectrums recorded during CNT-ME aptasensor fabrication. The spectrum (a) presents the Ag-Cysteamine, and the peak at 500 cm^−1^, corresponding to Ag-S stretching vibration, confirming the chemical attachment of cysteamine on the electrode surface [41]. The peaks at 800, 3360, and 1500 cm^−1^ corresponded to the C-H stretching vibration, the N-H, and NH_2_ stretching vibration.

The spectrum (b) displays the IR after immobilizing the COOH-MWCNTs. In particular, the peak at 1633 cm^−1^ can be assigned to the stretching of the C=C bond, while the peak at 1720 cm^−1^ can be corresponded to the C=O stretching vibration of R–COOH. Two peaks at 3350 cm^−1^ and 3348 cm^−1^ are attributed to H-O and N-H asymmetrical stretching vibration and symmetrical stretching vibration. Moreover, we noticed the disappearance of the NH_2_ stretching vibration at 1500 cm^−1^, which indicated that COOH-MWCNTs were chemically attached to the surface of the electrode. After the immobilization of the aptamer-MB, the IR spectrum (Figure S3c), the presence of the symmetric deformation of –CH_3_ at 1354 cm^−1^, symmetric stretching of C-N at 1398 cm^−1^ and the ring stretching of MB at 1603 cm^−1^, proves the absorption bands of MB on top of the CNTs. Moreover, the strong adsorption peak at 1671 cm^−1^ corresponding to the C=O formed at the interface COOH-MWCNTs and aptamer-MB. These results confirmed the successful immobilization of aptamer-MB on top of the COOH-MWCNTs, and therefore the attachment of the aptamers on the electrode surface. The results of CVs and EIS are consistent with the FTIR measurements, indicating the successful fabrication of the aptasensors (Figure 1).

Figure S4 shows the infrared spectrums (IR) recorded during AgNP-ME aptasensor fabrication. The spectrum (a) shows the Ag-1,8-Ocanedithiol, with a peak at 500 cm^−1^ corresponding to Ag-S stretching vibration. Moreover, the presence of the thiol group (S-H) was proved by the appearance of the absorbance peak at 2563 cm^−1^, which confirms the chemical attachment of 1,8-Ocanedithiol on the electrode surface.

The spectrum (b) displays the IR after immobilizing the AgNPs. The increase of the peak at 500 cm^−1^, which corresponds to Ag-S stretching vibration and the disappearance of the peak at 2563 cm^−1^ assigned for S-H, confirms that the AgNPs were covalently attached. 

After the immobilization of the aptamer-MB, the IR spectrum (Figure S4c), the appearance of MB absorption bands, such as the symmetric deformation of –CH_3_ at 1354 cm^−1^, the symmetric stretching of N-C at 1398 cm^−1^, and the ring stretching of MB at 1603 cm^−1^. These results confirmed the successful chemical attachment between AgNPs and the aptamers-MB, and therefore the attachment of the aptamers on the electrode surface. The results of CVs and EIS are consistent with the FTIR measurements, indicating the successful fabrication of the aptasensors (Figure 1).

### 3.3. Aptasensor Performance Comparison

#### 3.3.1. Electroactive area Evaluation of the CNT-ME and AgNP-ME

As shown in Figure S5, the oxidation and reduction current peaks (Ip) of the bare electrode in AgNP-ME and CNT-ME were proportional to the square root of the scan rate, which can be related to a reversible electrochemical behavior. Therefore, with the known parameters (D, C, and n), we can calculate the value of area (A) of the electrodes using the Randles–Sevcik Equation (supplementary material). Based on it, electroactive surface areas of 0.47 cm^2^, 1.01 cm^2^, and 1.79 cm^2^ for electrochemically cleaned bare electrode, AgNP-ME, and CNT-ME, were acquired, respectively. These results indicate how AgNPs and CNTs effectively enhanced the electroactive surface area of the electrode for the fabrication of an electrochemical biosensor. Due to the higher surface area to volume ratio, CNTs were chosen.

#### 3.3.2. Detection Performance

##### Optimization of Experimental Conditions

To attain better analytical performance, the important parameters of the experiment—such as amplitude and frequency of SWV, aptamers concentration, pH, incubation temperature, and incubation time—were optimized. The goal is to discover a set of parameters, which maximizes the current change before (hereinafter called ‘blank’) and after analyte usage.

Table S2 shows that the aptamers concentration immobilized onto the electrode had an influence on the performance of the aptasensors. When the number of aptamers was increased from 1 µM to 2 µM, the current change (i_pfuraneol_-i_pblank_) was also enhanced. After that, when the concentration of the immobilized aptamers was increased to 3, 4, and 5 µM, the blank current decreased, maybe due to the fact that the electrode surface was crowded and thus the MB has repelled away from the electrode surface. The maximum current change generated was when 2 µM and 1 µM of aptamers were immobilized onto CNT-ME and AgNP-ME, respectively.

Table S2 shows the anodic peak current as a function of different pH values of electrolyte (0.1 M PBS). The peak current change increased when the pH was increased, ranging from 4.0 to 7.0, and then decreased for higher pH values (from 8.0 to 11.0). Single-stranded (ss) DNA is a sequence of nucleotides. The most important force that links each ssDNA together to form double-stranded (ds) DNA and gives the aptamers their secondary structure is the hydrogenic bonds between cytidine (C)–guanosine (G) and adenosine (A)–thymidine (T). The formation of the hydrogen bond is strictly dependent on the pH. If the pH of the electrolyte is too low, the H^+^ in the surrounding environment of dsDNA is high, and therefore the hydrogen bonds between C-G and A-T will break in a competitive manner, which may induce a different aptamer arrangement and hence will decrease the affinity of the aptamer [42]. At high pH, the electrolyte is rich in hydroxide ions (negatively charged), these ions can disrupt the hydrogen bonding that gives the aptamers their structure, by pulling off hydrogen ions from the base pairs. Therefore, if the pH of the electrolyte is either lower or higher than the pH used during the aptamer isolation process, the aptamer will induce a change in its secondary structure, leading to a decrease in the affinity. In conclusion, pH = 7.4 was chosen for the entire voltammetry experiment. 

The effect of frequency on the peak current change in 0.1 M phosphate buffer solution at pH = 7.4 was studied by varying the frequency from 15 to 150 Hz (see Table S2). For both CNT-ME and AgNP-ME, the peak current was increased with increasing frequency up to 50 Hz and 75 Hz, respectively, and then the peak current became leveled off and decreased. Therefore, frequencies of 50 Hz and 75 Hz were chosen for subsequent experiments for CNT-ME and AgNP-ME, respectively.

The effect of square wave amplitude on the peak current of methylene in 0.1 M PBS of pH = 7.4 was studied, by increasing the amplitudes from 25 mV to 150 mV, as shown in Table S2. Upon increasing the amplitude, a linear increase in the peak current was observed until 50 mV; after that, the shape of the voltammogram lost cohesion. Hence, 50 mV was chosen as the amplitude for the next experiments for both modified electrodes.

The incubation temperature had an influence on the measured current. As shown in Figure S6, the aptasensor was incubated with different concentrations of furaneol (0, 50, 250, 500 pM) at 4 °C and room temperature. The one incubated at room temperature generated the highest currents, while at 4 °C generated lower current. This is because, the aptamers were isolated at room temperature, where they had the highest affinity towards the target.

Finally, the incubation time was studied. As shown in Table S2, for incubation times higher than 10 min, the current showed a constant value. For this reason, 10 min was chosen as the optimum time to switch from cDNA to furaneol. A summary of the optimized parameters used for the next experiments is shown in Table S3.

##### Comparison between AgNP-ME and CNT-ME Aptasensor

To compare which one of the two structures responds to a wider detection range, a series of furaneol concentrations were prepared (1 fM, 2 pM, 200 pM, 200 nM, and 10 µM). 

**Figure 4:**
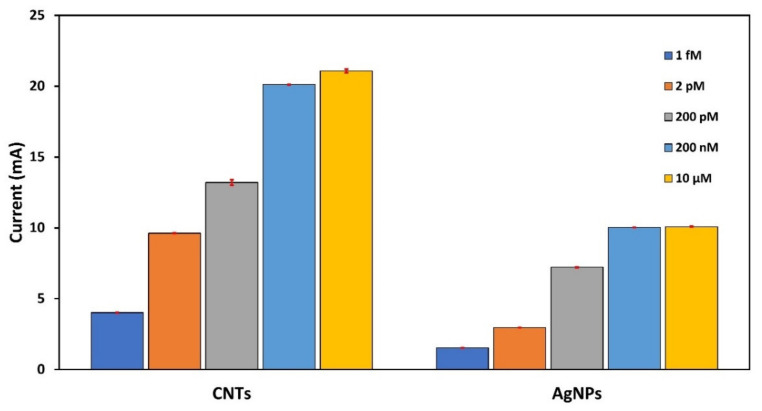
Comparison between CNT-ME/MB-aptamers and AgNP/MB-aptamers at different concentrations—1 fM, 2 pM, 200 pM, 200 nM, and 10 µM—of furaneol.

Figure 4 shows the aptasensor performances for both configurations (CNT-ME and AgNPs). The AgNP-ME sensor showed a current saturation starting from 200 nM; also, the inability to detect the concentration of 1 fM. In the other hand, the CNT-ME device was able to measure 1 fM–10 µM of the analyte. The reason for this may be the high electroactive area of CNT-ME compared to AgNP-ME (1.79 cm^2^ and 1.01 cm^2^, respectively), which allowed an immobilization of a high number of aptamers that increased the sensitivity of the aptasensor. Therefore, all the following experiments were performed only with the CNT-ME/MB-Aptamer.

### 3.4. CNT-ME Aptasensor

#### 3.4.1. Analytical Performance of the Aptasensor

**Figure 5:**
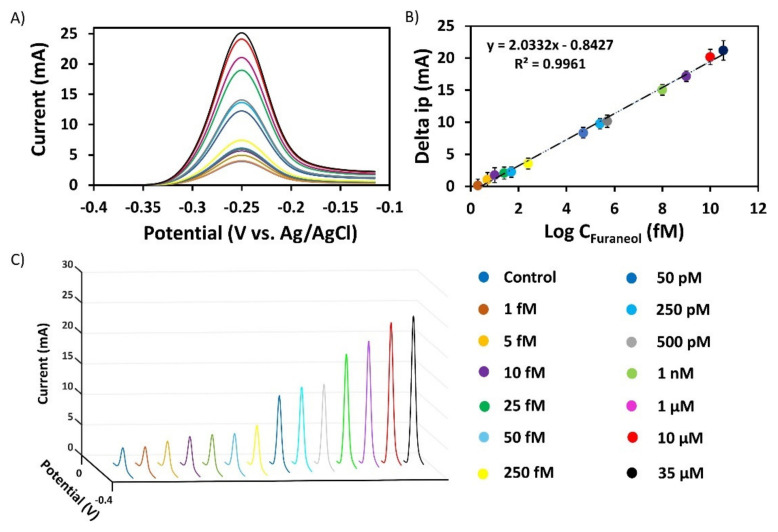
(**A**) and (**C**) Variation of the aptasensor response against the furaneol concentrations in the range of 1 fM–35 µM; (**B**) The linear relationship between the peak currents and furaneol concentration ranging from 1 fM to 35µM (n = 5).

The sensitivity of the aptasensor was evaluated with various concentrations of furaneol under the optimum measurement conditions (2 µM MB-Apt in immobilization buffer, Tris-HCl buffer pH 7.4, 50 V amplitude, 75 Hz frequency, incubation for 10 min at room temperature). Figure 4 shows the SWV CNT-based aptasensor responses to different furaneol concentrations (1 fM to 35 mM). The current peak of MB observed at −0.25 V increases accordingly, with increasing concentration of furaneol ranging from 1 fM to 35 μM (see Figure 5A,C). The relative current response of MB (Δi_p_) exhibits a good linear correlation with the logarithmic value of the target concentration (logC) as shown in Figure 4B. The correlated linear equation was Δi_p_ [mA] = (0.203) × logC [fM] − 0.842 with a determination coefficient (R2) = 99.61% (0.9961). The limit of detection was calculated from the calibration curve using Equation (1)

LOD = 3.3 STDEV I_p0_/m (1)

where STDEV I_p0_ is the standard deviation of the blank measurement and m is the slope, which is calculated using Equation (2)

m = (I_p1_ − I_p0_)/(C_1_ − C_0_) (2)

where I_p1_ is the current generated from concentration C_1_ (1 fM), and I_p0_ is the current generated from the C_0_ blank measurement.

Hence, LOD was calculated to be 0.557 fM. The wider detection range of the aptasensors may be due to three reasons. First, the higher conductivity of the silver ink compared to other inks (gold, carbon, etc.). Secondly, the enhancement of the electroactive surface area compared to the bare electrode, which was obtained by the CNTs modification. The enhancement of the electroactive area promoted the electron transfer between the electrolyte and the electrode surface and allowed a higher number of aptamers to be immobilized onto the working electrode, widening the linear detection range. Finally, the use of aptamers as sensing material/biorecognition elements, providing high affinity and selectivity for the specific analyte. Table 1 shows the developed biosensors for the detection of furaneol. The CNTs-ME enhanced the LOD, and the linear range compared to the field-effect transistor; however, the upper limit is low compared to the quartz crystal microbalance sensor. 

#### 3.4.2. Repeatability

For future commercial employment of the realized biosensors, the capability to be regenerated for multiple uses is a key aspect. For this reason, the repeatability of the aptasensors was investigated using different concentrations (1 fM, 250 pM, 10 µM) and optimized conditions. Reproducible SWV peaks were obtained with a relative standard deviation (RSD) of 3.16%, 3.46%, 2.0%, respectively (Table S4). This may be due to the use of the relative current response (subtracting the current generated from a blank sample) instead of taking directly the current generated, which may decrease the noise in the measurement and increase the signal/noise ratio. for instance, the difference in the number of aptamers immobilized on WE between one aptasensor and another may decrease the sensitivity. However, by subtracting the current generated from the blank sample, in this way, only the current generated because of the analyte will be measured, no matter the amount of the aptamers immobilized on the WE [26,27,28,29,30,31,32,33,34,35,36,37,38,39,40,41,42,43,44]. Hence, we increase the reproducibility of the aptasensors.

#### 3.4.3. Storage Stability and Regeneration Test

Another important parameter to investigate is the sensor stability in time to detect possible measurements drifts due to aging effects. The stability of the aptasensor was investigated by storing it at 4 °C and room temperature in dry condition and measuring a fixed concentration of 1 µM, every second day. Aptasensors stored at room temperature after two weeks retained about 78% of the initial response signal. Meanwhile, the aptasensor stored at 4 °C showed high signal retention of about 97% after two weeks and 93.26% after 25 days. The good time stability is mainly due to the stability of the aptamers and the SAM, which strengthened the immobilization of the aptamers onto the electrode surface (Figure S7).

In a similar way, a regeneration test was performed by detecting 1 µM of furaneol solution and subsequentially immersing in NaCl (5M) for 5 min and hybridizing the aptasensors with cDNA for 10 min. After five regenerations, a relative standard deviation (RSD) of 4.32% was obtained, proving the good regeneration ability of the aptasensors (Figure S8).

#### 3.4.4. Selectivity

**Figure 6:**
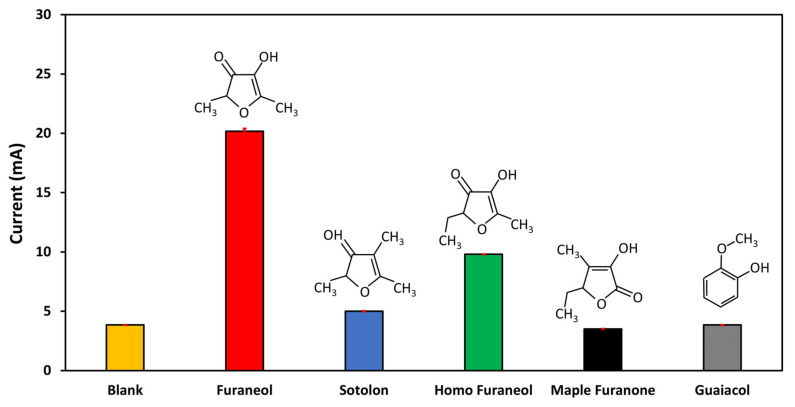
Test of the aptasensor for the detection of furaneol. The non-target analytes selected are sotolon, homo furaneol, maple furanone, and guaiacol. The concentrations of furaneol and non-target chemicals were 1 µM.

The aptasensor selectivity is an important parameter. To assess it, different analytes, which feature a similar chemical structure of furaneol, were detected with the same concentration and under the same conditions. For this experiment, sotolon, homo furaneol, maple furaneol, and guaiacol, were used. As shown in Figure 6 and Figure S9, the current signals were as follows 3.85 mA, 20.08 mA, 5.2 mA, 9.81 mA, 3.94 mA, and 3.84 mA for blank, furaneol, sotolon, homo furaneol, maple furanone, and guaiacol, respectively. The results show that current signals of the non-target compounds were almost the same as blank measurement, except for homo furaneol. This means no detection occurred, thanks to the good affinity of the implemented aptamers. These results are in agreement with previous work [19] with a bit of improvement for sotolon and maple furanone. This improvement may be due to the use of the CNTs, where a high number of aptamers were immobilized, moreover, it may be due to the optimization process where it was reported before that the surface density affects the affinity of the aptamers [45]. However, for homo furaneol, a weak current increase was observed. This is because both furaneol and homo furaneol have a very similar chemical structure, which makes the selectivity complex. Nevertheless, furaneol has an I_p_ current value of 112% higher than that of the homo furaneol.

#### 3.4.5. Mechanical Stability Test

Adopting high throughput and low-cost mass production fabrication processes to reduce the production cost is important for commercial viability. In this context, the roll-to-roll (R2R) fabrication process is a promising solution for sensor fabrication; however, the use of a flexible substrate (e.g., PET) is mandatory [46]. Hence for a successful adaptation of flexible, not rigid substrates in biosensing, we need to evaluate the stability of the final product (aptasensor) under mechanical stress. At this aim, the aptasensor flexibility was tested by measuring a concentration of 1 µM furaneol, while bending the device for multiple times (50, 100, 250, 500, 1000, 1500, 2000, and 3000 bending cycles) down to a bending radius of 6 mm. The current generated after each set of bending cycles was evaluated (Figure S10). An increase of RSD with the increasing number of cycles was noticed, maybe due to the increase of the mechanical stress, which led to the detachments of the aptamers and cracking of the electrodes, decreasing the current flow. To clarify whether the aptasensor performance degradation depends on the biorecognition elements or on the materials employed for the electrodes, another experiment was performed, in which a CNT-ME without aptamers was bent for the same number of cycles, measuring the resistance of the electrode after each bending cycle. The resistance increased with the increased bending cycles number with an RSD of 1.18%, 1.18%, 1.21%, 1.31%, 2.73%, 2.98%, 3.38%, and 3.96%. As presented in Figure S10, the RSD values of the CNT-ME are lower than the aptasensor, which may indicate that mechanical stress has more effect on the detachment of the aptamers than on CNT-ME. Nevertheless, the aptasensor showed good stability even after 3000 bending cycles, and this is most likely due to the SAM, which made the aptasensor layers very stable for the stress applied.

#### 3.4.6. Detection of Furaneol in Real Samples and HPLC Validation

**Figure 7:**
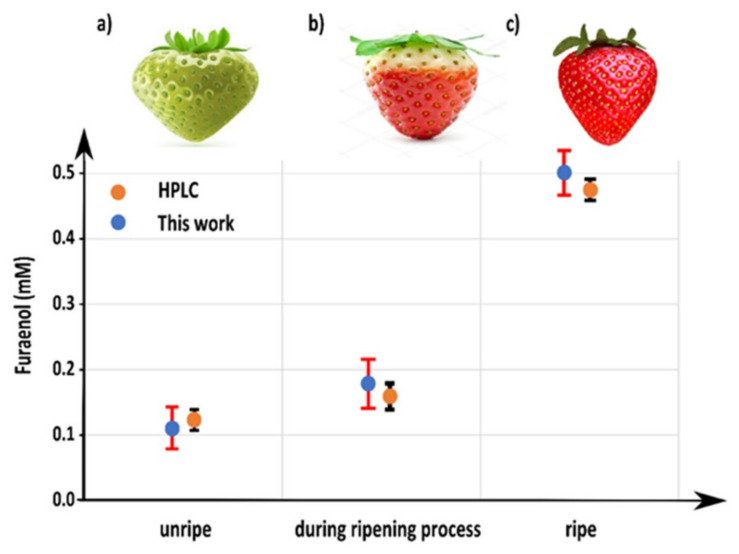
Validation of the aptasensor with HPLC by measuring furaneol in a real sample: (**a**) unripe strawberry, (**b**) during ripening process strawberry, and (**c**) ripe strawberry (tested samples n = 3).

To examine the effect of the real sample matrix on the aptasensor performance and to validate the aptasensor, the following experiment was performed. Figure 7 displays the amount of furaneol found in different maturation stages (n = 3) (unripe, during the ripening process, ripe) of Elsanta strawberry (Figure S11) measured using HPLC and the developed aptasensor. The results showed an increase in the furaneol from unripe (113.7 µM) during the ripening process (166.75 µM), and ripe (481.14 µM), which is in agreement with the results previously found [8]. The aptasensor was in good agreement with those of HPLC (Figure 6), with average recoveries of 97.66%, 106%, and 108.16% and with the coefficient of variance ranging from 3.8%, 4.9%, and 9.6% for unripe, ripening, and ripe, respectively. This test indicates that the unripe strawberry matrix effect was almost negligible on the performance of the aptasensor. However, after this stage, the effect of the matrix was noticeable, where higher concentrations were measured, and this may be due to the presence of furaneol glucoside, mesifurane, and homo-furaneol as well as the aptasensor low sensitivity towards them. Hence, the measured concentration was higher than the real one [8]. Also, it is a proof of the good reliability and accuracy of the developed aptasensor.

## 4. Conclusions

In summary, a flexible printed electrochemical aptasensor was developed for furaneol detection and functionalized with two different nanomaterials (CNTs and AgNPs). The deposition of the CNTs and AgNPs increased the electroactive area of the working electrode from 0.47 cm^2^ (bare electrode) to 1.01 cm_2_ and 1.79 cm^2^ for AgNP-ME and CNT-ME, respectively. The CNT-MEs have a higher electroactive area than the AgNP-MEs, which may be due to the higher surface area to volume ratio. This increase in the electroactive area enhanced the sensitivity of the aptasensor by allowing immobilization of a higher number of aptamers. The CNT-modified aptasensor showed a wider detection range from 1 fM to 35 µM and a lower LOD of 0.55 fM, while AgNP-ME aptasensors had a detection range from 2 pM to 200 nM. However, in an application where CNTs could affect the measurement such as gas sensing, AgNPs could be a great alternative. Furthermore, the CNT-ME aptasensor showed high reproducibility with RSD of 3.16%, fast measurement time (10 min), storage stability (25 days at 4 °C), selectivity, and mechanical stability. Moreover, a validation test was performed to evaluate furaneol concentration in real samples (Elsanta strawberry) with both HPLC and the developed aptasensor, showing good accuracy between 5% and 12%. The reported aptasensor has many potential applications in the food industry, such as controlling the process during food production, food product origin, beverage quality, or controlling the ripeness of fruits. 

## Figures and Tables

**Table 1:** Comparison of the sensitivity of the developed biosensors for the detection of furaneol

Sensor	Biorecognition Element	Linear Range	LOD	Reference
Field Effect Transistor	Aptamers	0.1–10 µM	0.1 µM	[19]
Quartz crystal microbalance	Molecular imprinted polymer	780.46–7804.57 µM	9.6 µM	[43]
Square Wave Voltammetry	Aptamers	1 fM–35 µM	0.557 fM	This work

## Supplementary Materials

The following are available online at https://www.mdpi.com/2079-4991/10/6/1167/s1, Figure S1: Screen-printed flexible electrodes; Figure S2: Characterization of the aptasensor stability under mechanical stress (bending); Figure S3: FT-IR spectra of (a) Ag-Cysteamine (in black), (b) Ag-Cysteamine-COOH-MWCNT (in orange), (c) Ag- Cysteamine-COOH-MWCNT-Aptamer-MB (in blue); Figure S4: FT-IR spectra of (a) Ag-1,8-Octanedithiol (in black), (b) Ag-1,8-Octanedithiol-AgNP (in red), (c) Ag-1,8-Octanedithiol-AgNP -Aptamer-MB (in blue); Figure S5: (A), (C), and (E) are CVs of bare electrode, AgNP-ME and CNT-ME respectively; Figure S6: Effects of incubation temperature on the current signals generated from CNT-ME (A) and AgNP-ME (B); Figure S7. Time stability test for CNT-ME aptasensor stored at 4 °C and 21 °C (n = 5). Figure S8: Regeneration test of the CNT-ME aptasensor printed for furaneol detection (n = 5); Figure S9: Selectivity test of the aptasensor for the detection of furaneol; Figure S10: Bending test of the CNT-ME aptasensor printed on PET for furaneol detection (n = 5); Figure S11: Elsanta strawberries extract from different maturation stages; Table S1: EIS characterization of the modified electrodes during the different process steps; Table S2: Effect of MB-Apt concentration, electrolyte pH, frequency, amplitude and incubation time on the current signals generated from CNT-ME (A) and AgNP-ME (B). Corresponding to blank (without furaneol), post analyte (with 250 pM of furaneol), and ΔI (current change caused by 250 pM of furaneol) (n = 5). Table S3: Optimized values of the parameters (n = 10).
