# Supplementary material for: Flexible Screen Printed Aptasensor for Rapid Detection of Furaneol: A Comparison of CNTs and AgNPs Effect on Aptasensor Performance
Source: Nanomaterials (Basel). 2020 Jun 15;10(6):1167. doi: 10.3390/nano10061167 (PMC7353281; doi:10.3390/nano10061167)
Supplement: Supplementary file 1 [file nanomaterials-10-01167-s001.pdf]

Flexible Screen Printed Aptasensor for Rapid Detection of Furaneol: A Comparison of CNTs and AgNPs Effect on Aptasensor Performance

Ali Douaki *, Biresaw Demelash Abera, Giuseppe Cantarella, Bajramshahe Shkodra, Asma Mushtaq, Pietro Ibba, AKM Sarwar Inam, Luisa Petti *, and Paolo Lugli

Faculty of Science and Technology, Free University of Bolzano-Bozen, 39100 Bolzano, Italy; BiresawDemelash.Abera@natec.unibz.it (B.D.A.); Giuseppe.Cantarella@unibz.it (G.C.); Bajramshahe.Shkodra@natec.unibz.it (B.S.); Asma.Mushtaq@natec.unibz.it (A.M.); Pietro.Ibba@natec.unibz.it (P.I.); AKMSarwar.Inam@natec.unibz.it (AKM.S.I.); Paolo.Lugli@unibz.it (P.L.)

* Correspondence: adouaki@unibz.it (A.D.); Luisa.Petti@unibz.it (L.P.)

Received: 21 May 2020; Accepted: 12 June 2020; Published: date

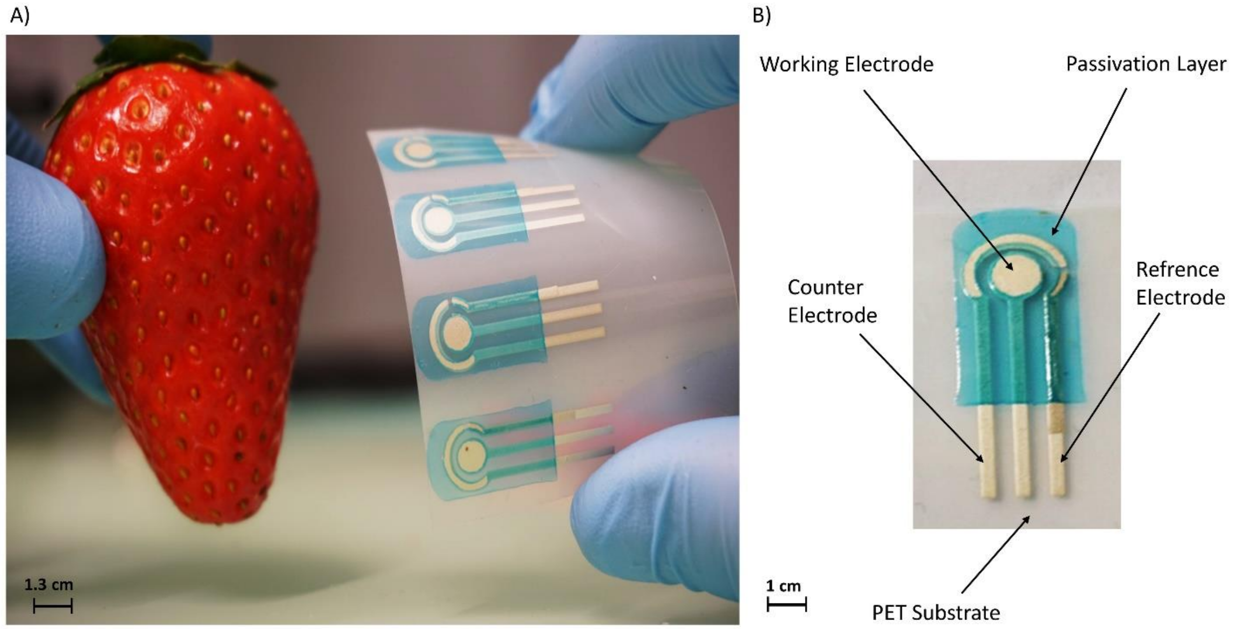

Figure S1. Screen-printed flexible electrodes: (**A**) aptasensor beside a strawberry (scale bar: 1.3 cm) and (**B**) electrode device schematic (scale bar: 1 cm).

1. Mechanical characterization setup:

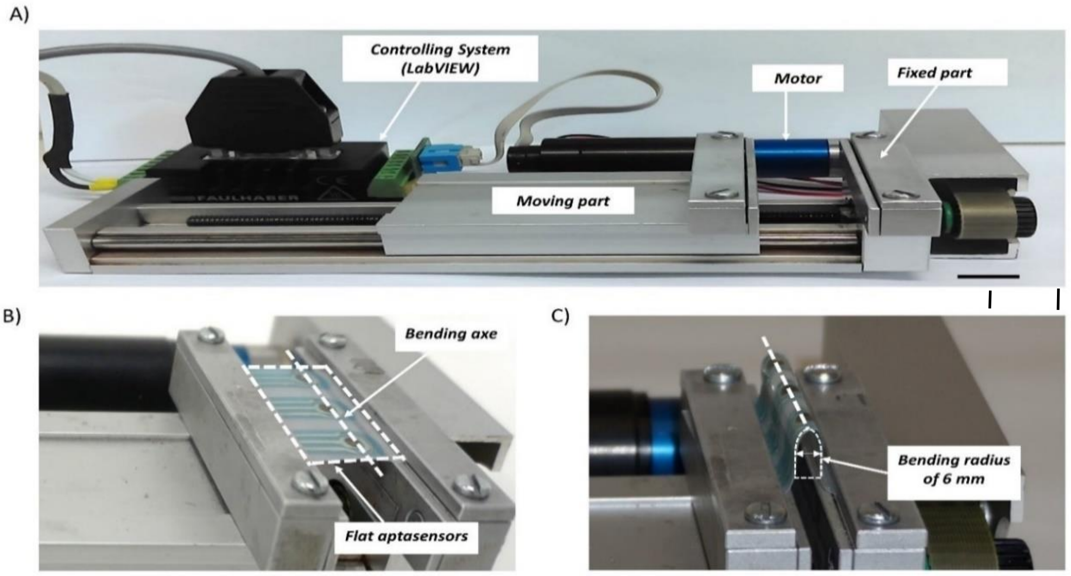

Figure S2. Characterization of the aptasensor stability under mechanical stress (bending), (**A**) flat aptasensors, (**B**) bent aptasensors (with a radius of 6 mm), and (**C**) custom-made bending-setup controlled using a LabVIEW program (Scale bar: 1 cm).

2. EIS characterization of electrodes:

Table 1. EIS characterization of the modified electrodes during the different process steps

	**CNT modified electrodes R_{ct} (Ω)**	**AgNPs modified electrodes R_{ct} (Ω)**
Bare	40	43
Bare/nanomaterial	32	24
Bare/nanomaterial/Aptamers	1956	1238
Bare/nanomaterial/Aptamers/cDNA	1705	1005

3. FTIR Characterization:

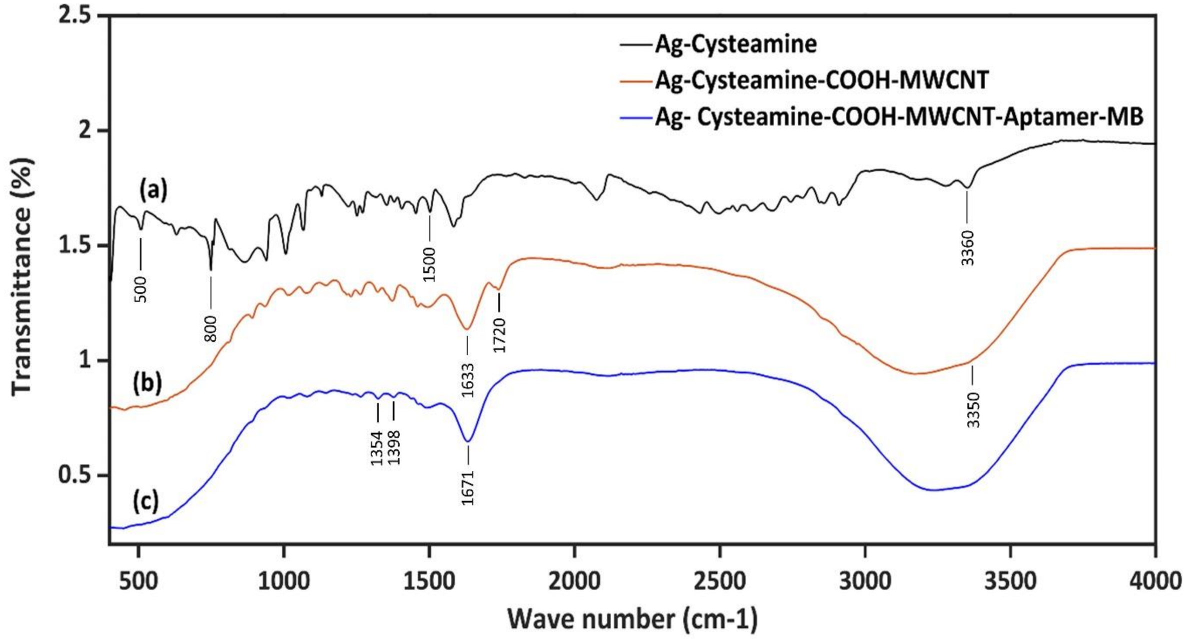

Figure S3. FT-IR spectra of (**a**) Ag-Cysteamine (in black), (**b**) Ag-Cysteamine-COOH-MWCNT (in orange), (**c**) Ag-Cysteamine-COOH-MWCNT-Aptamer-MB (in blue).

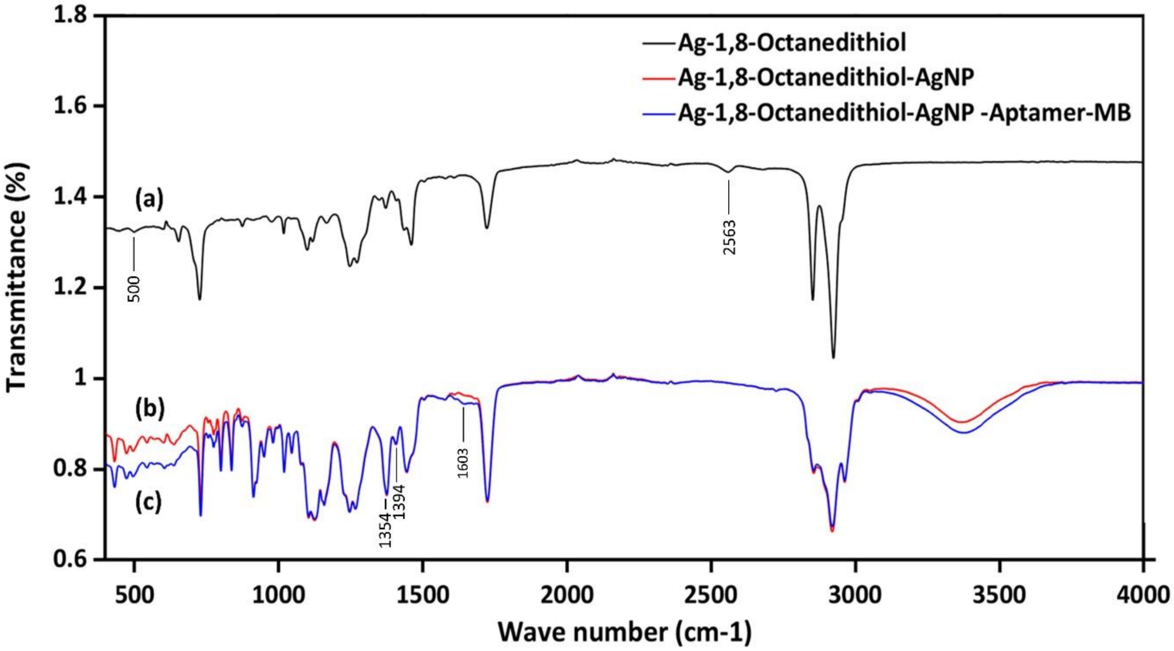

Figure S4. FT-IR spectra of (**a**) Ag-1,8-Octanedithiol (in black), (**b**) Ag-1,8-Octanedithiol-AgNP (in red), (**c**) Ag-1,8-Octanedithiol-AgNP -Aptamer-MB (in blue).

4. Electro-active area evaluation of the Ag/CNTs and Ag/AgNPs

In order to check the effect of nanomaterials on the electroactive surface area of the electrodes, the latter was evaluated by means of cyclic voltammetry. The measurements were performed in 5 mM [Fe (CN)6]3-/4- solution containing 0.1 M KCl with scan rates ranging from 10 to 200 mV/s. Thus, according to the obtained cyclic voltammograms (CVs), the electroactive surface area of the bare, AgNPs-ME and CNTs-ME can be estimated based on Randles–Sevcik equation $I_P = 2.69\times10^5$ A D1/2 $n^{3/2}$ $v^{1/2}$ C [1], in which I_P refers to the peak current of CV, A is the electro-active surface area (cm2), D is the diffusion coefficient of $[Fe(CN)^6]^{3-/4-}$ (at 25 °C, D = 6.7×10^{-6} cm^2 s^{-1}), n is the number of the electrons transferred in the reaction (n=1), v is the scan rate (V s^{-1}), and C is the concentration of the reagent (5x10^{-6} mM).

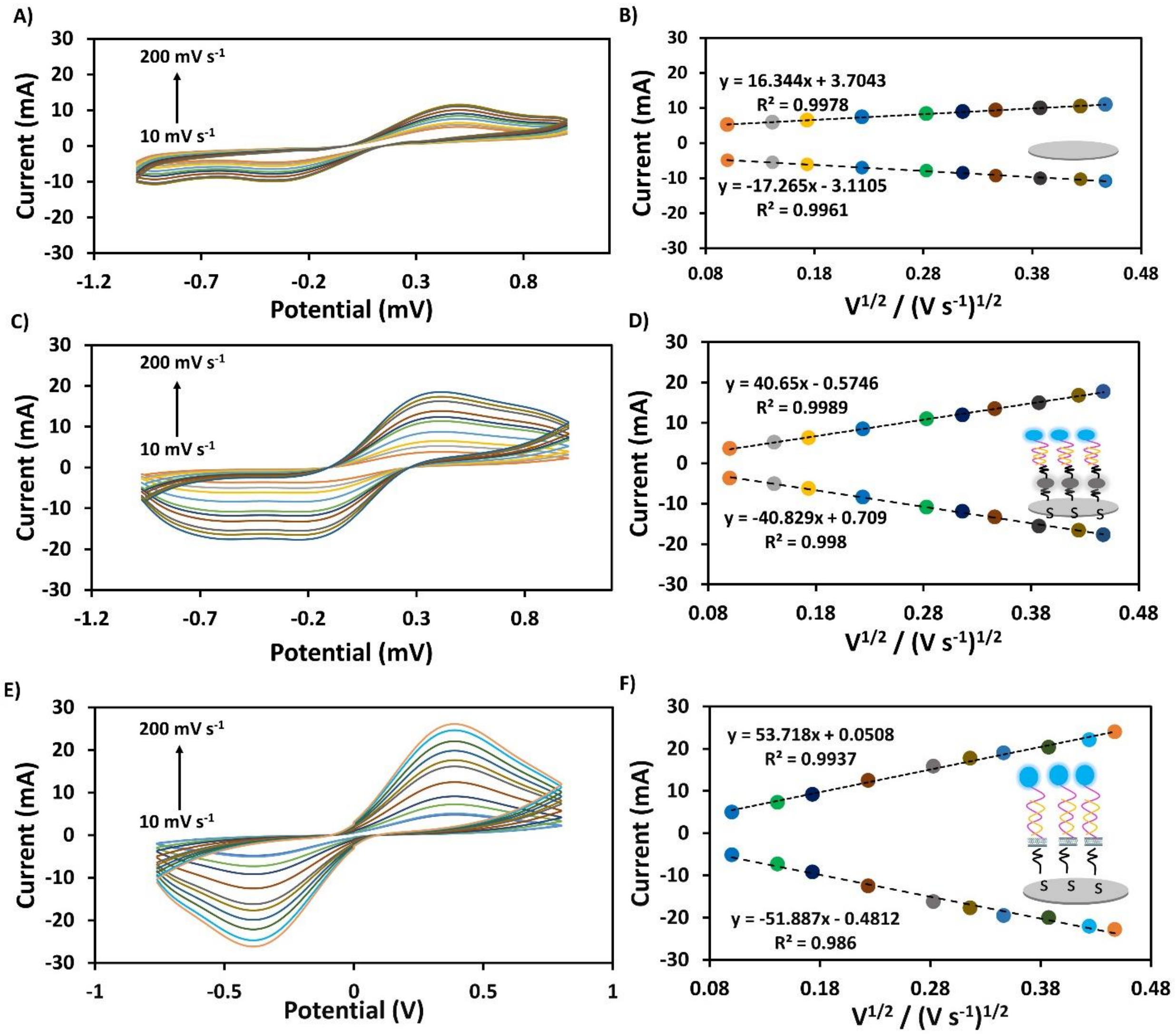

Figure S5. (**A**), (**C**), and (**E**) are CVs of bare electrode, AgNP-ME and CNT-ME respectively in 5.0 mM [Fe(CN)6]3-/4- containing 0.1 M KCl at various scan rates (10, 20, 30, 40, 50, 80, 100, 120, 150, 180, 200 mV s−1). (**B**), (**D**), and (**F**) Plots of the oxidation and reduction peak currents vs. square root of scan rates of bare electrode, AgNP-ME and CNT-ME, respectively.

5. Optimization of experimental conditions:

Table S2. Effect of MB-Apt concentration, electrolyte pH, frequency, amplitude and incubation time on the current signals generated from CNT-ME (A) and AgNP-ME (B). Corresponding to blank (without furaneol), post analyte (with 250 pM of furaneol), and ΔI (current change caused by 250 pM of furaneol) (n=5).

		**Blank (mA)**		**Post (mA)**		**ΔI (mA)**	
		**CNTs**	**AgNPs**	**CNTs**	**AgNPs**	**CNTs**	**AgNPs**
Concentration (μM)	0.5	2.128 ± 0.22	1.006 ± 0.18	5.264 ± 0.15	5.029 ± 0.26	3.136 ± 0.35	4.024 ± 0.37
	1	2.86 ± 0.1	1.52 ± 0.12	9.226 ± 0.19	7.511 ± 0.08	6.366 ± 0.15	5.986 ± 0.19
	2	4.022 ± 0.13	1.722 ± 0.14	14.214 ± 0.15	6.035 ± 0.1	10.192 ± 0.23	4.312 ± 0.18
	3	4.426 ± 0.15	1.936 ± 0.13	9.774 ± 0.08	5.266 ± 0.25	5.348 ± 0.17	3.337 ± 0.14
	4	4.97 ± 0.12	2.248 ± 0.23	9.778 ± 0.13	4.654 ± 0.23	4.808 ± 0.19	2.403 ± 0.23
	5	5.374 ± 0.11	2.552 ± 0.32	7.246 ± 0.11	4.012 ± 0.1	1.872 ± 0.17	1.454 ± 0.27
pH	4	1.214 ± 0.11	0.83 ±0.12	1.644 ± 0.18	2.126 ± 0.2	0.43 ± 0.23	1.3 ± 0.2
	5	1.318 ± 0.13	1.115 ± 0.08	2.224 ± 0.13	3.521 ± 0.13	0.906 ± 0.23	2.422 ± 0.2
	6	2.364 ± 0.15	1.336 ± 0.1	7.864 ± 0.25	5.424 ± 0.11	5.56 ± 0.27	4.09 ± 0.15
	7	4.104 ± 0.11	1.454 ± 0.14	14.246 ± 0.2	7.455 ± 0.35	10.142 ± 0.21	5.95 ± 0.34
	8	3.572 ± 0.13	1.336 ± 0.2	7.338 ± 0.13	6.452 ± 0.29	3.766 ± 0.18	5.112 ± 0.37
	9	3.022 ± 0.23	1.32 ± 0.07	7.464 ± 0.15	5.243 ± 0.2	4.442 ± 0.23	3.91 ± 0.24
	10	2.46 ± 0.21	1.266 ± 0.17	2.89 ± 0.15	4.227 ± 0.25	0.43 ± 0.02	2.948 ± 0.41
	11	1.976 ± 0.2	1.013 ± 0.21	2.134 ± 0.21	2.25 ± 0.26	0.158 ± 0.03	1.248 ± 0.44
Frequency (Hz)	25	3.45 ± 0.15	1.012 ± 0.07	9.02 ± 0.15	4.21 ± 0.05	5.546 ± 0.07	3.204 ± 0.11
	50	3.84 ± 0.18	1.245 ± 0.19	13.836 ± 0.3	5.853 ± 0.16	9.955 ± 0.39	4.612 ± 0.33
	75	5.648 ± 0.22	1.538 ± 0.08	15.366 ± 0.53	7.24 ± 0.08	9.681 ± 0.62	5.727 ± 0.1
	100	6.438 ± 0.16	2.227 ± 0.16	15.75 ± 0.28	7.644 ± 0.19	9.352 ± 0.38	5.42 ± 0.3
	125	7.152 ± 0.21	2.921 ± 0.2	16.428 ± 0.22	8.127 ± 0.22	9.256 ± 0.2	5.19 ± 0.23
	150	7.866 ± 0.3	3.513 ± 0.16	17.634 ± 0.27	8.666 ± 0.05	9.72 ± 0.33	5.167 ± 0.15

	5	3.458 ± 0.13	0.943 ± 0.07	7.22 ± 0.23	4.502 ± 0.08	3.762 ± 0.26	3.571 ± 0.04
	25	3.984 ± 0.08	1.1322 ± 0.13	9.554 ± 0.13	6.229 ± 0.13	5.57 ± 0.12	5.073 ± 0.14
	50	4.214 ± 0.11	1.437 ± 0.17	14.412 ± 0.13	7.434 ± 0.25	10.198 ± 0.16	6.036 ± 0.39
Amplitude (mV)	75	5.372 ± 0.08	1.518 ± 0.14	14.228 ± 0.04	6.857 ± 0.21	8.856 ± 0.05	5.37 ± 0.23
	100	5.754 ± 0.18	1.766 ± 0.19	12.75 ± 0.07	6.14 ± 0.29	6.996 ± 0.18	4.438 ± 0.42
	125	5.862 ± 0.1	1.922 ± 0.13	9.236 ± 0.2	5.742 ± 0.27	3.374 ± 0.19	3.89 ± 0.14
	150	6.076 ± 0.15	2.216 ± 0.18	8.952 ± 0.31	4.563 ± 0.27	2.876 ± 0.37	2.372 ± 0.35
	5	3.75 ± 0.1	1.568 ± 0.15	8.66 ± 0.23	5.532 ± 0.22	4.96 ± 0.2	3.943 ± 0.19
	10	3.846 ± 0.12	1.552 ± 0.25	13.644 ± 0.18	7.531 ± 0.08	9.817 ± 0.18	5.986 ± 0.24
Time (min)	15	3.657 ± 0.07	1.604 ± 0.21	13.852 ± 0.21	7.565 ± 0.3	10.231 ± 0.21	5.911 ± 0.42
	20	4.018 ± 0.13	1.54 ± 0.25	13.748 ± 0.19	7.58 ± 0.24	9.72 ± 0.29	6.057 ± 0.2
	25	3.948 ± 0.08	1.526 ± 0.16	13.646 ± 0.16	7.516 ± 0.1	9.67 ± 0.17	5.985 ± 0.21
	30	3.852 ± 0.08	1.592 ± 0.13	13.666 ± 0.18	7.571 ± 0.17	9.812 ± 0.16	6.35 ± 0.15

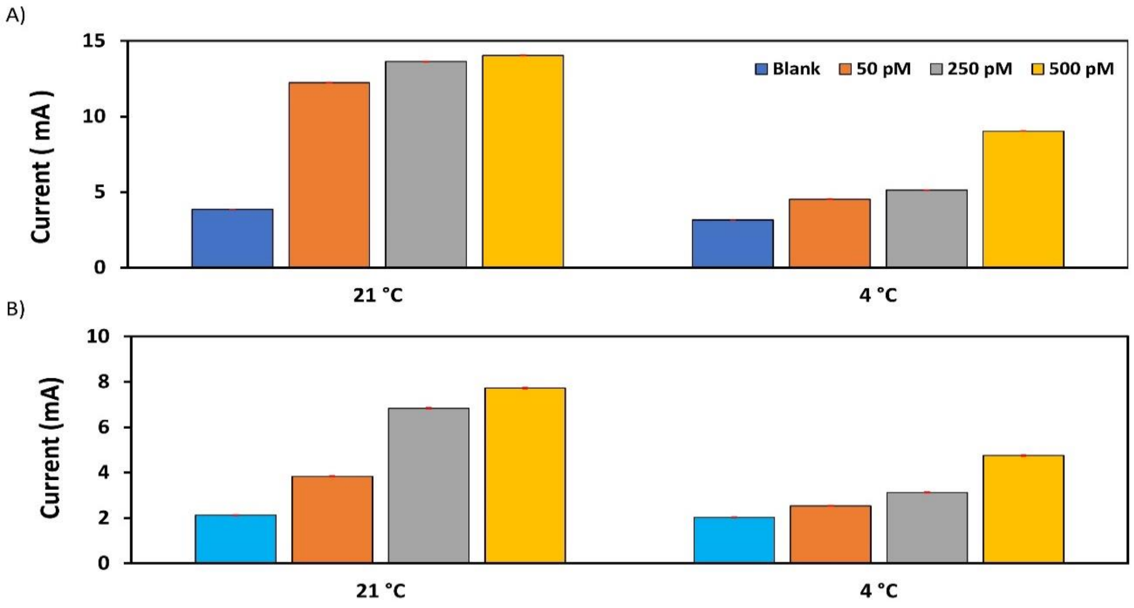

Figure S6. Effects of incubation temperature on the current signals generated from CNT-ME (**A**) and AgNP-ME (**B**). Corresponding to blank (without furaneol), post analyte (with 250 pM of furaneol), and ΔI (current change caused by 250 pM of furaneol). in E-buffer (pH = 7.4), [1 μM and 2 μM MB-Apt in immobilization buffer for AgNP-ME and CNT-ME, respectively], frequency [75 (Hz) and 50 (Hz) for AgNP-ME and CNT-ME, respectively], amplitude (50 mV) and incubation for 10 min (n = 5).

Table S3. Optimized values of the parameters

Parameters	AgNP modified electrode	CNT modified electrode
Aptamer Concentration (μM)	1	2
Amplitude (mV)	50	50
Frequency (Hz)	75	50
pH	7	7
Incubation Temperature (°C)	21	21
Incubation Time (min)	10	10

6. Repeatability

Table S4: Optimized values of the parameters (n = 10)

	1 fM	200 pM	10 μM
ΔI current (mA)	0.154	9.65	20.02
Relative standard deviation	3.16%	3.46%	2%

7. Regeneration test and storage stability

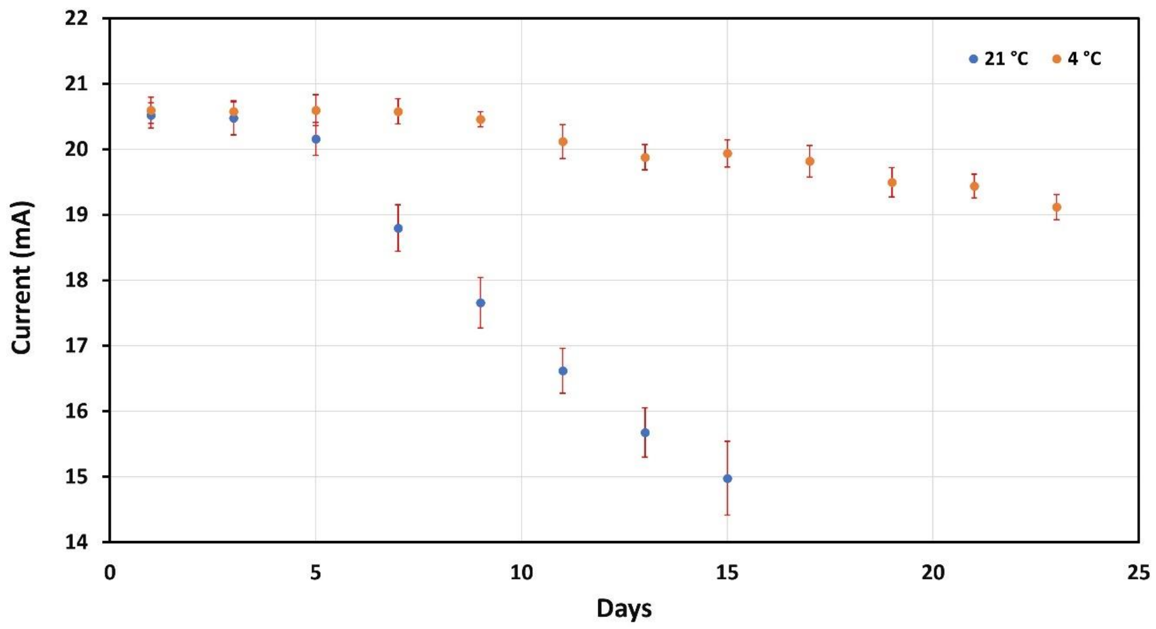

Figure S7. Time stability test for CNT-ME aptasensor stored at 4 °C and 21°C (n = 5).

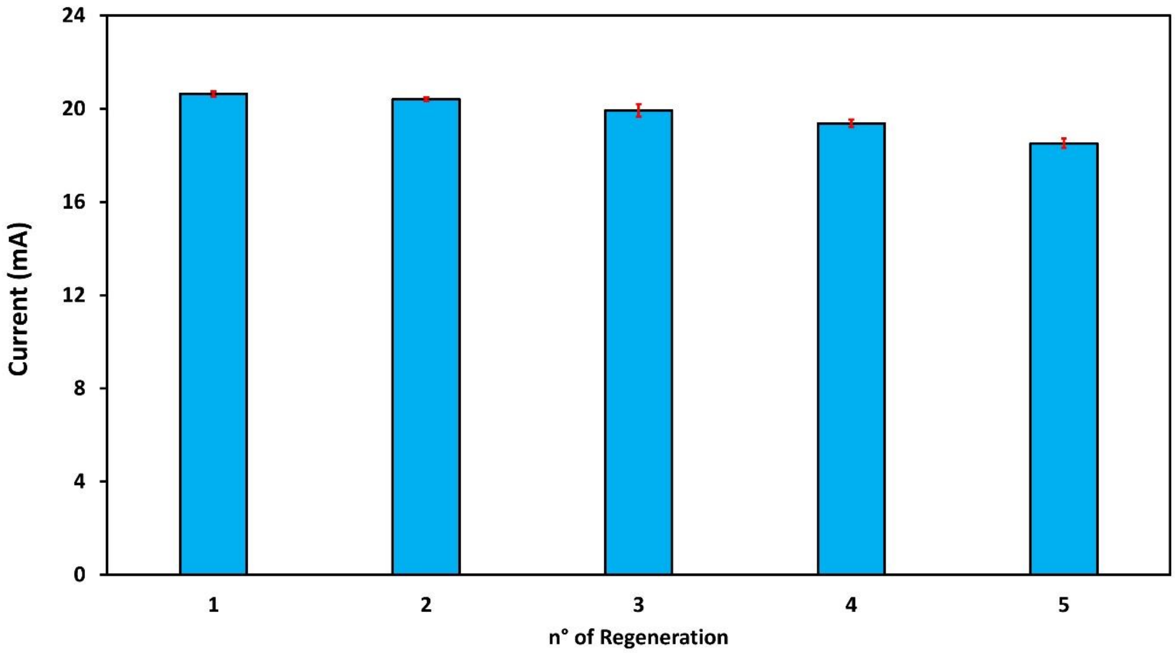

Figure S8. Regeneration test of the CNT-ME aptasensor printed for furaneol detection (n = 5).

8. Selectivity:

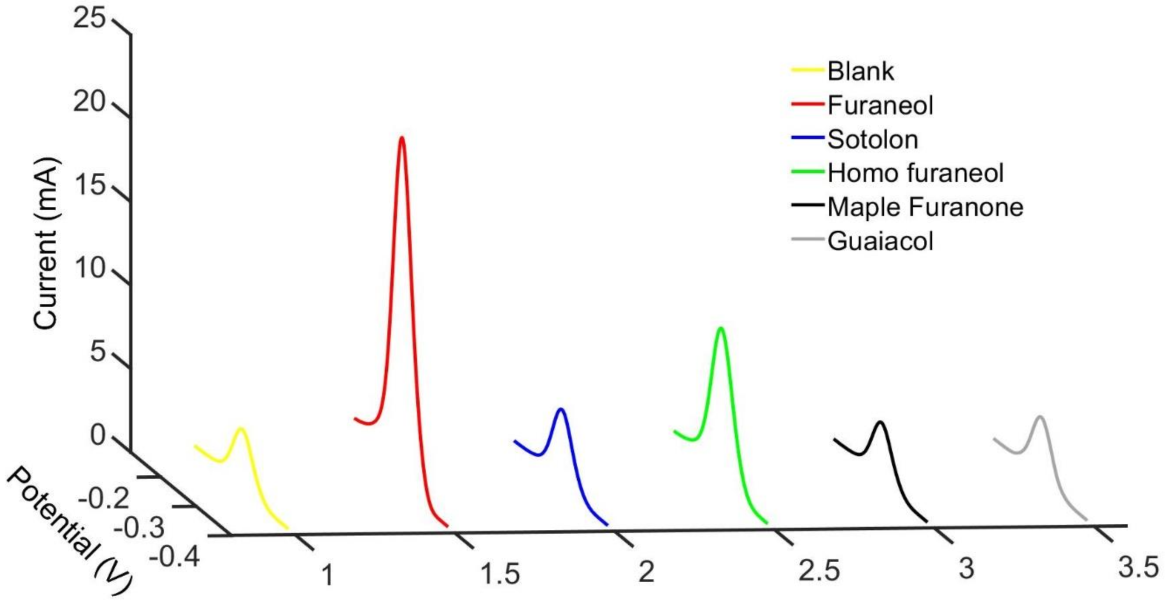

Figure S9. Selectivity test of the aptasensor for the detection of furaneol. The non-target analytes selected are sotolon, homo furaneol, maple furanone, guaiacol. The concentrations of furaneol and non-target chemicals were 1 μM.

9. Mechanical characterization

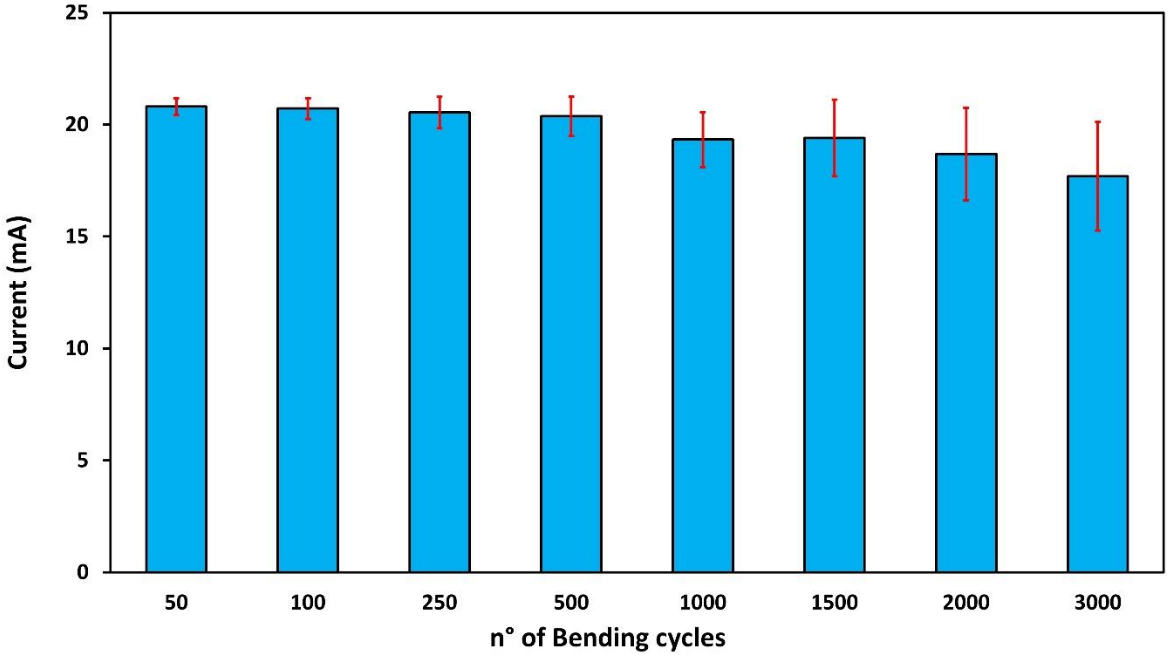

Figure S10. Bending test of the CNT-ME aptasensor printed on PET for furaneol detection (n = 5).

10. Detection of furaneol in a real sample and validation with HPLC

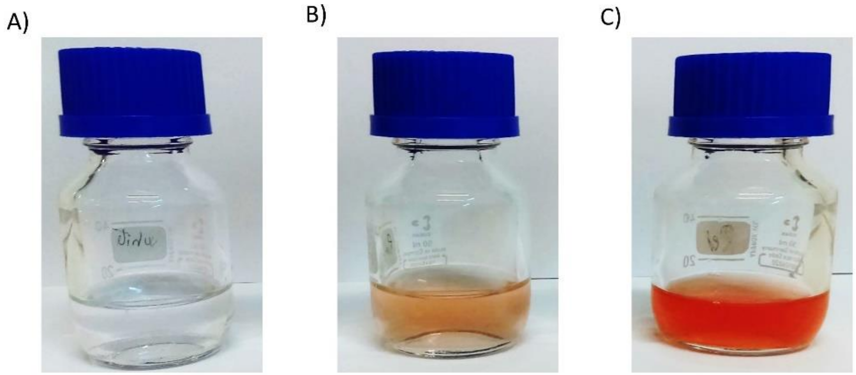

Figure S11. Elsanta strawberries extract from different maturation stages, (**A**) unripe, (**B**) during the ripening process, and (**C**) ripe.

References

1. Ferrari, G.; Foster, C.C.; Kelly, P.J.; Brownson, D.A.C.; Banks, C.E. Determination of the Electrochemical Area of Screen-Printed Electrochemical Sensing Platforms. Biosens, 2018, 8, 53.
